# Determination of Phylogroups, Pathotypes and Antibiotic Resistance Profiles of *E. coli* Isolates from Freshwater and Wastewater in the City of Panama

**DOI:** 10.3390/pathogens14070617

**Published:** 2025-06-20

**Authors:** Gabriela A. Rodríguez Guevara, Emmanuel Michelangelli, Juan R. Medina-Sánchez, Fermín Mejía-Meléndez, Carmen Indira Espino, José E. Moreno P., Alex O. Martínez Torres, Jordi Querol-Audí

**Affiliations:** 1 Laboratory of Experimental and Applied Microbiology, Universidad de Panamá, Panama City 7096, Panama; gabriela98alexa@gmail.com (G.A.R.G.); emmanuelmc997@gmail.com (E.M.); jmr2095@gmail.com (J.R.M.-S.); fermejia25@gmail.com (F.M.-M.); 2 Department of Human Microbiology, Faculty of Medicine, Universidad de Panamá, Panama City 7096, Panama; carmen.espino@up.ac.pa; 3 Bioscience and Biotechnology Doctorate, Technological University of Panamá, Panama City 7095, Panama; josmoresearch@gmail.com; 4 Sistema Nacional de Investigación (SNI), SENACYT, Panama City 7098, Panama; 5 Department of Biochemistry and Nutrition, Faculty of Medicine, Universidad de Panamá, Panama City 7096, Panama

**Keywords:** *E. coli*, antibiotic resistance, phylogroups, pathotypes, freshwater, wastewater

## Abstract

Untreated water bodies are critical ecological niches where environmental conditions can drive the adaptive evolution of bacterial populations, enabling them to acquire new traits such as antibiotic-resistance genes. *Escherichia coli* is typically a commensal bacterium but can evolve into a pathogenic form, known as Diarrheagenic *E. coli*, responsible for both intestinal and extraintestinal diseases. This study focuses on the characterization of *E. coli* isolates from water samples collected from the Matasnillo River and the influence of the Juan Díaz Wastewater Treatment Plant (WWTP). While isolates from the Matasnillo River were classified as commensal, 18% of the isolates from the WWTP belonged to either phylogroups D or B2. Pathotype analysis revealed the presence of Entero-Toxigenic and Entero-Hemorrhagic *E. coli* in the WWTP. Moreover, Matasnillo River isolates exhibited resistance mainly to the quinolone ciprofloxacin, whereas those from the WWTP influent showed resistance to multiple broad-spectrum antibiotics. Sequencing analysis revealed the prevalence of the transmissible quinolone resistance *qnrB19* among the Matasnillo River isolates and mutations conferring resistance to quinolone in *gyrA*, *parC*, and *parE*. These findings highlight the importance of monitoring antibiotic-resistant bacterial contamination in both freshwater and wastewater to mitigate the risk of the spread of resistant pathogens and potential epidemic outbreaks.

## 1. Introduction

Waterborne pathogens are a major global health threat, causing over 2.2 million deaths annually, particularly affecting vulnerable populations, such as children under five and the elderly. Contaminated water sources are often reservoirs for a range of infectious agents, including *Salmonella* sp., *Shigella* sp., *Giardia lamblia*, *Vibrio cholerae*, *Cryptosporidium parvum*, enteropathogenic strains of *Escherichia coli*, and enteric viruses [1]. Diarrheal diseases, frequently associated with unsafe drinking water and poor sanitation infrastructure, represent the second leading cause of death in children under five years globally, with an estimated 525,000 fatalities each year [2]. In low-resource settings, where access to clean water and sanitation facilities remains limited, contaminated waters continue to be a key driver of preventable illness and death [3].

*E. coli* is widely used as an important indicator of fecal contamination in water sources, thus the identification of pathogenic strains is useful in predicting human health risks [4]. Consequently, phylogroup classification is widely used to understand the evolutionary relationships and pathogenic potential of *E. coli* isolates [5]. Despite their high diversity, commensal and extraintestinal pathogenic *E. coli* are not phylogenetically separated and the transition to infection is complex [6]. While commensal *E. coli* populations are prevalent within their host, specific subpopulations exhibit pathogenicity in both humans and animals [7].

Pathogenic *E. coli* is a highly diverse group of bacteria that can also be classified into several pathotypes based on their virulence factors, mechanisms of infection, host interactions, and clinical manifestations. These include enteropathogenic (*EPEC*), Shiga toxin-producing (*STEC*), enteroaggregative (*EAEC*), enterotoxigenic (*ETEC*), enteroinvasive (*EIEC*), and diffusely adherent (*DAEC*) *E. coli* [8]. These strains can cause a wide range of diseases, both intestinal (e.g., diarrhea, dysentery) and extra-intestinal (e.g., urinary tract infections, neonatal meningitis). Diarrheagenic *E. coli* (DEC) strains, in particular, are responsible for a significant proportion—up to 30–40%—of acute diarrheal episodes in children, especially in low- and middle-income countries, where sanitation and hygiene infrastructure is often inadequate [9,10,11]. While the primary mode of transmission is fecal-oral, contamination of water sources by DEC strains further amplifies the risk of disease outbreaks. The persistence of these pathotypes in water systems highlights their potential to spread rapidly.

In Latin America, a high prevalence of different DEC pathotypes has been reported, along with their distribution in each country, especially those present in various water bodies such as rivers, reservoirs, and wastewater treatment influent plants [12]. Even after treatment, effluents from WWTPs often contain high concentrations of these pathogens, which are released into receiving bodies of water that are typically used for irrigation (agricultural, parks, and gardens) and non-potable urban uses (e.g., irrigation of green areas, toilets, and washing) [13]. In fact, several studies have linked the presence of EPEC in contaminated waters to outbreaks of acute diarrhea due to food contamination [14,15,16]. In Latin American countries such as Brazil, Argentina, and Ecuador, EPEC strains were found in domestic animals (including chicken, pigs, lambs, and dogs) and its presence was associated with diarrhea in children [12]. DEC strains, particularly those associated with acute diarrhea, are increasingly exhibiting multidrug resistance, e.g., up to 50% of cases showed resistance to multiple antibiotics in Mexico and Ethiopia [17,18,19] and multidrug resistance DEC prevalence ranged between 18.5% and 97.1% in Asian countries [20,21]. This issue is part of a broader global problem, largely driven by the overuse and misuse of antibiotics in both healthcare and agriculture [22]. Furthermore, the lack of new antibiotic development has worsened the spread of antimicrobial resistance, making it a significant public health threat worldwide.

Given the lack of reports in Panama on environmental pathogenic and antibiotic-resistant *E. coli,* which could represent a risk to public health, this study aimed to characterize and classify *E. coli* isolates from two main water sources. One of the sources is the Matasnillo River, a 6 km long inner-city river in Panama City. This river traverses from the north of Panama City drains into the Panama Bay and is subject to pollutants from both industrial and urban environments. The other source is raw wastewater the influent of the Wastewater Treatment Plant of the City (known as the Juan Díaz Wastewater Treatment Plant, WWTP), which receives and treats wastewater from around one million of the city’s inhabitants using aerobic treatment, nutrients removal, sludge treatment, and biogas production systems. By comparing the distribution of phylogroups, pathotypes and antibiotic resistance profiles of *E. coli* isolates between highly polluted waters (wastewater at the influent of the WWTP) and freshwater (Matasnillo River), the main objective of this research was to evaluate the microbiological quality of the Matasnillo River in order to establish whether circulating waters in the City could represent a potential threat to the population.

## 2. Materials and Methods

### 2.1. Sample Collection

This study used a cross-sectional design to gather information regarding antibiotic resistance profiles among *E. coli* isolates from two different water sources in Panama City. Water samples (1 L each) were collected once in each source as a first screening for environmental and wastewater evaluation.

A surface water sample was collected at the Matasnillo River mouth (Paseo Cinta Costera, Paitilla, Panama City, Panama. 8°58′28.6″ N; 79°31′6.2″ W) in a sterilized glass bottle in the direction of the current. The sample was transported refrigerated to the laboratory and processing was conducted upon arrival (less than 24 h).

Additionally, a collection of 17 cryo-preserved *E. coli* isolates obtained from a wastewater sample from the influent at the WWTP of Panama City was provided by the Experimental and Applied Microbiology Laboratory of the University of Panama (Universidad de Panama). The same experimental procedure for the isolation and characterization of *E. coli* from both samples was used (see Section 2.2 and Section 2.3).

### 2.2. Isolation of E. coli

The isolation of *E. coli* strains was performed following the protocol established in the Standard Methods for the Examination of Water and Wastewater [23]. Briefly, 1 mL of the water sample was inoculated into 10 mL of Lauryl Tryptose Broth and Durham hood (a total of 10 tubes) and incubated at 37 °C for 24 h. Cultures that showed gas production and turbidity were inoculated into 10 mL of Brilliant Green Bile Broth and Durham hood and incubated at 44.5 °C for 24 h. The media that showed turbidity and gas formation were then streaked onto EMB agar and incubated at 37 °C for 24 h. Colonies that presented a green color with a metallic sheen and a dark center were incubated in EC-MUG broth and Durham hoods at 44.5 °C for 24 h to detect β-Glucuronidase activity. Those that presented turbidity, gas formation, and fluorescence under UV light were considered positive for the presence of *E. coli*. After streaking these cultures on nutrient agar and incubating at 37 °C for 18 h, isolated colonies were inoculated in nutrient broth and incubated for 18 h at 37 °C. The resulting cultures were cryopreserved in 20% glycerol at −80 °C until use.

### 2.3. Characterization of E. coli Isolates

The VITEK 2 System^®^ (Biomérieux^®^, Marcy-l’Étoile, France) was used for bacterial confirmation and antimicrobial susceptibility profile determination, following the protocol established by the manufacturer. Briefly, the isolates were inoculated into nutrient broth and incubated at 37 °C for 24 h. Streaks were then performed on LB agar plates, which were incubated at the same temperature for 18 h. Colony-forming units (CFU) were then transferred using a sterile swab to tubes containing sterile 0.85% saline solution, until an optical density of 0.50–0.63 McFarland was reached. The bacterial suspensions were introduced onto the GN (Gram-negative) cards for bacterial identification and AST cards for antimicrobial susceptibility testing (Biomérieux^®^, Marcy-l’Étoile, France) and incubated in the VITEK 2 COMPACT^®^. Identification results and resistance profiling were pre-analyzed by the Advance Expert System (AES Expert, 2.0.0 version).

### 2.4. DNA Extraction

DNA was extracted using the boiling method. Briefly, *E. coli* cells were resuspended into nuclease-free water until 0.5 McFarland was reached. The samples were boiled for 10 min and centrifugated at 10,000× *g* for 10 min. A supernatant containing the DNA was recovered. DNA concentration and purity were determined using a NanoDrop^TM^ 2000/2000c Spectrophotometer (Thermo Fisher Scientific, Waltham, MA, USA).

### 2.5. Phylogenetic Group Determination of the E. coli Isolates

Phylogenetic groups were determined by PCR targeting the *yja*A, *chu*A, and *Tsp*E4C2 genes and using the dichotomous Clermont key, as previously described [24]. PCR mixtures contained 10 µL of 2X AmpliTaq Gold^TM^ 360 Master Mix (Thermo Fisher Scientific, Waltham, MA, USA), sample DNA (50 ng), and 0.5 µM of each forward and reverse primer, in a final volume of 20 µL. PCR conditions for target genes were as follows: denaturation for 5 min at 94 °C; 30 cycles of 30 s at 94 °C, 30 s at 55 °C, and 30 s at 72 °C; and a final extension step of 5 min at 72 °C.

### 2.6. Determination of Pathotypes of E. coli Isolates

Pathotyping was performed by two multiplex PCR protocols, based on the amplification of specific genes previously described [25]. Multiplex was carried out using AmpliTaq Gold^TM^ 360 Master Mix (Thermo Fisher Scientific, Waltham, MA, USA), 0.2 µM of each primer, 100 ng template DNA, in a final volume of 20 µL. Multiplex conditions were as follows: initial denaturation at 95 °C for 5 min, followed by 35 cycles of: denaturation at 95 °C for 30 s, annealing at 60 °C [for multiplex 1 and 55 °C for multiplex 2] for 30 s, extension at 72 °C for 30 s; and a final extension at 72 °C for 5 min. PCR products were run in a 1.5% agarose gel in 1X TBE buffer and visualized under UV light. Target genes for each multiplex and their respective pathotype are listed in Table 1.

### 2.7. Detection of Quinolone-Resistance Mechanisms

PCR was used to amplify known regions of *gyrA*, *parC,* and *parE* as previously described [26,27]. PCR conditions were as follows: denaturation for 5 min at 94 °C; 30 cycles of 30 s at 94 °C, 30 s at 55 °C for *gyrA* [56 °C for *parC* and 58 °C for *parE*], and 1 min at 72 °C; and a final extension step of 5 min at 72 °C. Amplicon sequences were obtained by Sanger sequencing (Psomagen, Rockville, MD, USA). Chromosomal mutations conferring resistance to quinolones were analyzed by aligning the amino acid sequences with the reference *E. coli* strain K-12 sub. MG1655. Aligning plots were made using the R package ggmsa v1.13.1 [28].

Additionally, PCR and sequencing were used to detect and characterize quinolone resistance genes *qnrB*, *qnrS*, and *aac(6′)-Ib-cr* using specific primers [29,30]. PCR conditions were as follows: denaturation for 5 min at 94 °C; 30 cycles of 30 s at 94 °C, 30 s at 53 °C for *qnrB* and *qnrS* [60 °C for *aac(6′)-Ib-cr*], and 1 min at 72 °C; and a final extension step of 5 min at 72 °C. DNA sequences were compared with the GenBank database to subtype the antibiotic-resistance genes.

## 3. Results

### 3.1. Identification of Phylogroups and Pathotypes

**Figure 1:**
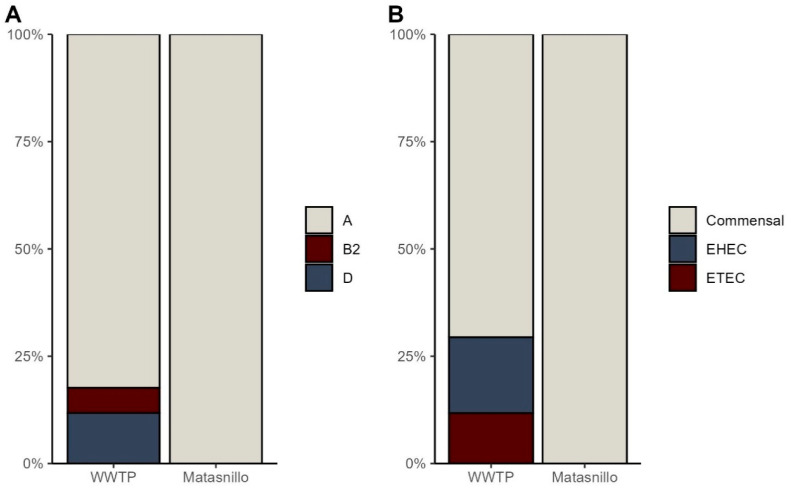
*E. coli* phylogroups (**A**) and pathotypes (**B**) from Juan Díaz WWTP and Matasnillo River.

The results showed that all strains isolated from the Matasnillo River (100%, 37/37) belonged to phylogroup A, which is predominantly composed of commensal bacteria while 18% (3/17) of the strains isolated from wastewater samples amplified the *chuA* gene (Supplementary Figure S1) and were further categorized as strains belonging to phylogroup D (12%, 2/17 isolates) and phylogroup B2 (6%. 1/17 isolates), which are both predominantly pathogenic bacteria. The rest of the isolates (72%; 14/17) belonged to phylogroup A (Figure 1A).

All the strains isolated from the Matasnillo River (37/37) were classified as commensal, as none of the virulence factors were amplified from those samples. In contrast, *est*B1 gene was detected in 2 out of the 27 strains isolated from the Juan Diaz WWTP (12%), indicating the presence of *ETEC* (Enterotoxigenic *Escherichia coli*), 18% (3/17) amplified the *stx*1 gene corresponding to *EHEC* (Enterohemorrhagic *Escherichia coli*), and 70% (12/17) were commensal (Figure 1B, Supplementary Figure S2).

### 3.2. Antibiotic Resistance Profiles

**Figure 2:**
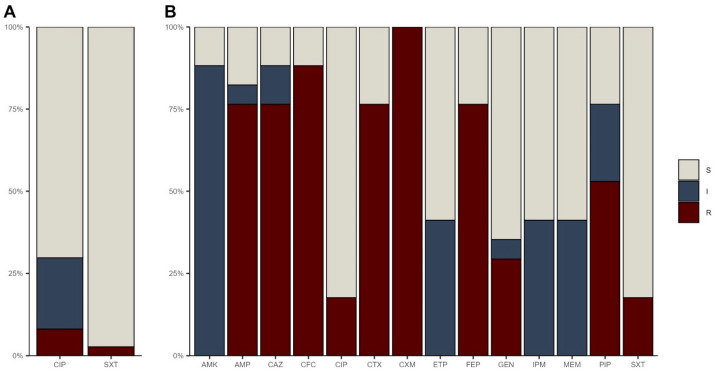
Proportion of antibiotic resistance phenotypes from *E. coli* strains isolated from (**A**) the Matasnillo River and (**B**) the Juan Diaz Wastewater Treatment Plant (WWTP): susceptible (S), intermediate (I), and resistant (R). Antibiotics: amikacin (AMK), ampicillin (AMP), ceftazidime (CAZ), cefazolin (CFC), ciprofloxacin (CIP), cefotaxime (CTX), cefuroxime (CXM), ertapenem (ETP), cefepime (FEP), gentamicin (GEN), imipenem (IPM), meropenem (MEM), piperacillin-tazobactam (PIP) and trimethoprim-sulfamethoxazole (SXT).

Antibiotic resistance was found in 32% (12/37) of isolates from the Matasnillo River. Resistance to ciprofloxacin was exhibited in 8% (3/37) of isolates, followed by 3% (1/37) to trimethoprim/sulfamethoxazole. Additionally, 22% (8/37) of the isolates displayed intermediate resistance to ciprofloxacin (Figure 2A).

The isolates from the WWTP showed resistance to the following antibiotics: 100% (17/17) to cefuroxime, 88% (15/17) to cefazolin, 76% (13/17) to ampicillin, ceftazidime, cefotaxime, and cefepime, 53% (9/17) to piperacillin-tazobactam, 29% (5/17) to gentamicin, 18% (3/17) to ciprofloxacin, and 18% (3/17) to trimethoprim/sulfamethoxazole. Furthermore, 88% (15/17) exhibited intermediate resistance, including 88% (15/17) to amikacin, 41% (7/17) to ertapenem, meropenem, and imipenem, 23% (4/17) to piperacillin-tazobactam, 12% (2/17) to ceftazidime, 6% (1/17) to gentamicin, and 6% (1/17) to ampicillin (Figure 2B).

#### 3.2.1. Multidrug Resistance Profiles

**Figure 3:**
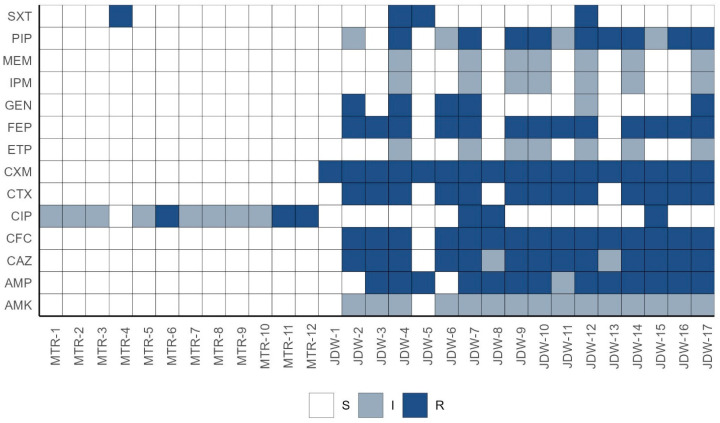
Antibiotic resistance profiles of *E. coli* isolates from the Matasnillo River (MTR-1 to MTR-12) and Juan Diaz Wastewater Treatment Plant (JDW-1 to JDW-17). The color fill indicates the resistance phenotype: S = susceptible, I = intermediate, and R = resistant.

No multidrug-resistant bacteria were detected among the isolates from the Matasnillo River, following the antimicrobial susceptibility profile determination used in this study. In contrast, 94% (16/17) of the *E. coli* isolates from the WWTP exhibited multidrug resistance (Figure 3), which included resistance to beta-lactams (cephalosporins) and aminoglycosides in combination with quinolones, sulfonamides and carbapenems.

The total of quinolone-resistant isolates belonged to phylogroup A. All the river quinolone-resistant isolates were commensal while 2 isolates from the WWTP sample were ETEC.

#### 3.2.2. Quinolone Resistance Mechanisms

Molecular detection revealed that 50% (7/14) of quinolone-resistant strains carried the *qnrB* gene (Supplementary Figure S3). All *qnrB*-positive isolates came from the Matasnillo River. The allele typing analysis of *qnrB* positives determined the prevalence of allele *qnrB19*. No other *qnrB* allele was found and *aac(6′)-Ib-cr* and *qnrS* alleles were not detected in any of the quinolone-resistant isolates.

**Figure 4:**
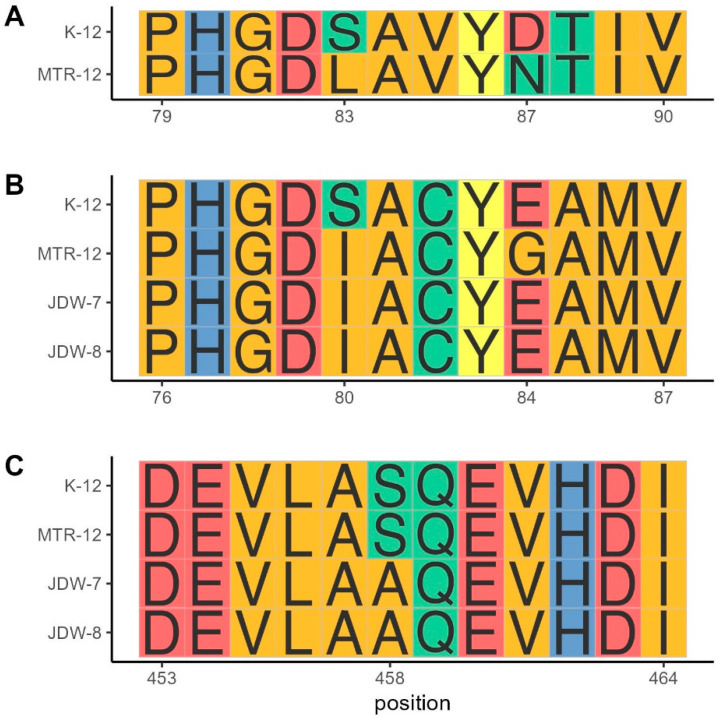
Amino acids alignments of (**A**) *gyrA*, (**B**) *parC*, and (**C**) *parE* regions and identified mutations conferring resistance to quinolones. Amino acids are colored according to their side-chain chemistry.

Mutations analysis revealed that 1 river isolate (MTR-12) presented *gyrA* mutations S83L and D87N (Figure 4A) and *parC* mutations S80I and E84G (Figure 4B). Two WWTP isolates (JDW-7 and JDW-8) presented both the same *parC* mutation (S80I, Figure 4B) and *parE* mutation (S458A, Figure 4C), even though *gyrA* sequences for these isolates could not be retrieved.

## 4. Discussion

This study focused on the characterization of *Escherichia coli* isolates obtained from untreated water sources, aiming to determine their antimicrobial resistance profiles and their phylogenetic and pathogenic groups. A high prevalence of multidrug-resistant isolates was found in the wastewater sample and a distinct quinolone resistance pattern was identified from the Matasnillo River isolates. Furthermore, pathogenic *E. coli* groups were only detected among wastewater isolates.

In this study, phylogroup A was predominant among the *E. coli* isolates from both water samples comprising all the isolates from the Matasnillo River and most isolates from the wastewater sample. This phylogroup is highly associated with human sources and most strains are commensal [31]. These findings are consistent with previous characterization of *E. coli* isolated from fecal-contaminated water sources and sewage [32,33].

Phylogroup A was predominant among the isolates from wastewater and lower but similar proportions of phylogroup D and B2 were detected. This distribution is a worldwide pattern, where phylogroup A is the most common followed by phylogroups D and B2, which usually have overlapping or low-frequency differences across locations [34]. Although phylogroup B2 and D are considered pathogens, phylogroup A is generally considered to represent commensal strains. It is important to note that some isolates within the phylogroup can carry virulence factors and cause disease, including urinary tract infections [35]. The differences between the distribution of virulence factors and phylogenetic groups may be related to geographical differences [36]. Therefore, the presence of commensal strains with quinolone resistance in the Matasnillo River indicates fecal contamination from humans or animals.

We investigated the presence of five clinically significant *E. coli* pathotypes—EHEC, EPEC, EIEC, ETEC, and DAEC—due to their relevance to public health and their association with outbreaks in Latin America [12]. Currently, little is known about the prevalence of circulating *E. coli* pathotypes in Panama. Publications about this topic were not found. Furthermore, the presence of pathogens in Panamanian water sources was not determined or published before. All isolates from the Matasnillo River were classified as commensal as none of the targeted virulence factors were detected in these isolates. Nevertheless, commensal *E. coli* is a potential reservoir for antibiotic resistance genes that can be transferred to other bacteria in the environment and within the host gut [37,38,39], thus continued monitoring of antibiotic resistance in Matasnillo River is crucial to manage and mitigate the spread of resistances in the city.

Despite the prevalence of commensal strains in the raw wastewater sample, EHEC and ETEC pathotypes were detected, posing a risk of infection for the WWTP workers and surrounding populations due to exposure to aerosolized bacteria. Both pathotypes remain important pathogens in Latin American countries. For example, EHEC infections in Argentina were a leading cause of acute renal failure among children and ETEC was associated with diarrheal cases in Mexico, Colombia, and Nicaragua [12]. Although the Shiga toxin producer EHEC-serotype O157 is worldwide predominant [40], the isolation methods suggest that the mentioned EHEC isolates do not belong to this serotype or are atypical O157:H7 since all exhibited β-Glucuronidase-positive phenotype and carried the *stx1* gene, as previously reported [41].

Antibiotic resistance in bacteria is a pressing global issue, with members of the *Enterobacteriaceae* family being particularly concerned. In this study, *E. coli* isolates from both wastewater and the Matasnillo River exhibited varying resistance profiles. A clear difference in resistance distribution was observed between the two samples where wastewater isolates demonstrated resistance to a broader range of antibiotic families compared to those from river water. Interestingly, both wastewater and river isolates showed resistance to the fluoroquinolone/quinolone antibiotic class. This aligns with clinical surveillance reports from Panama, which highlight fluoroquinolone resistance as particularly widespread among *E. coli* isolates [42]. Thus, the detection of quinolone-resistant and intermediately resistant isolates in the Matasnillo River underscores the impact of anthropogenic pollution on environmental bacterial populations. This situation increases the risk of waterborne infections and facilitates the dissemination of antibiotic-resistance genes between humans, animals, and the environment, which, again, highlights the importance of continuous microbiological monitoring of surface waters.

Quinolone as well as fluoroquinolones antibiotics and by-products have been detected worldwide in surface waters due to their wide use in human and veterinary medicine along with their limited absorption and excretion into the environment [43]. The presence of this antibiotic class increases the evolution of bacteria and generates resistance as a consequence of selection pressure [44] and can also induce resistance to non-quinolone antibiotics by increasing mutation rates, mutation frequencies, and recombination [45]. The presence of quinolone-resistant bacteria in the Matasnillo River may indicate the presence of these compounds even though the concentration has not been determined, posing a major health risk.

Quinolone resistance in Matasnillo River isolates is primarily conferred by quinolone resistance genes. Previous studies determined that *E. coli* isolates from aquatic environments are sources of plasmid-mediated quinolone resistance mechanisms such as *qnrS*, *qnrB*, *aac(6′)-Ib-cr*, *oqxAB* and *qepA* [46]. Most studies reported a high prevalence of *qnrS* in *E. coli* isolates from surface water sources used for drinking, irrigation, and recreational activities [47,48,49]. However, a high prevalence of *qnrB* was detected among the Matasnillo River isolates while *qnrS* was not detected. Our findings suggest that the Matasnillo River is a reservoir for *qnrB* genes and support previous reports proposing that the distribution of predominant quinolone-resistance mechanisms varies over geographical regions [50,51,52]. In this study, the allele *qnrB19* is prevalent within the quinolone resistance isolates from the Matasnillo River. In Latin American countries, the same allele was highly prevalent and mainly carried by commensal *E. coli* and *Salmonella* spp. isolated from human and water samples [53,54,55]. A similar prevalence of *qnrB19* was documented in different geographical regions including Enterobacteria isolated from free-living animals, stock, and food samples [56,57,58]. Moreover, identical *qnrB19*-carrying plasmids have been found in unrelated *Salmonella* serotypes and *E. coli*, suggesting the dissemination of quinolone resistance through horizontal transfer mechanisms [59]. Furthermore, plasmid transmission can lead to chromosome stabilization via transposition as previously reported in chromosomal *qnrB19*-carrying *E. coli* isolates [60].

Quinolone-resistance genes *qnrB* and *qnrS* were not detected in wastewater isolates. On the contrary, chromosomal mutations in *parC* and *parE* genes were identified. Comparably, chromosomal mutations in *gyrA*, *parC*, and *parE* genes have been reported as the primary cause of quinolone resistance rather than resistance genes in *E. coli* isolated from wastewater samples [61]. Wastewater isolates also exhibited resistance to cephalosporins, which suggests the production of cephalosporinases or extended-spectrum beta-lactamases (ESBL). High rates of ESBL-producing *Escherichia coli* resistant to quinolones have been also reported from samples collected from wastewater treatment plant influents [62]. Even though this study used a cross-sectional design as a first screening for antibiotic resistance profiles among environmental and wastewater *E. coli* isolates, our results emphasize the importance of continuous monitoring of the microbiological quality of circulating waters in Panama City.

## 5. Conclusions

This study has determined the presence of pathogenic and antibiotic-resistant *E. coli* strains in circulating waters in Panama City, highlighting the need for routine surveillance and stricter monitoring of microbial contaminants in wastewater before environmental discharge.

The isolation, identification, and characterization of *E. coli* pathotypes are important indicators of contamination when considering water quality. Furthermore, the study of the resistance profiles of these environmental strains is highly relevant for the ongoing monitoring of our surrounding environments, based on the one-health concept.

We recommend conducting periodic studies to assess the evolution of circulating resistant strains that impact public health. We should also consider comparing environmental monitoring isolates with clinical information to establish state actions to curb antibiotic resistance.

## Figures and Tables

**Table 1:** Pathotype multiplex PCR targeting genes.

Pathotype	Gene Target	Multiplex PCR 1	Multiplex PCR 2
EPEC	*eae*, *bfpB*	*eae*, *bfpB*	
EHEC	*eae*, *stx1*, *stx2*	*stx2*	*stx1*
EIEC	*virF*, *ipaH*	*virF*	*ipaH*
DAEC	*daaE*	*daaE*	
ETEC	*estB1*		*estB1*

## Data Availability

All the data that supports the findings of this study are available from the corresponding author J.Q.A, upon reasonable request.

## Supplementary Materials

The following supporting information can be downloaded at: https://www.mdpi.com/article/10.3390/pathogens14070617/s1, Figure S1: Detection of *chuA* gene; Figure S2: Results of Multiplex PCR 2 for detection of EHEC, EIEC and ETEC pathotypes; Figure S3: PCR detection of *qnrB* group.
