# Supplementary material for: Determination of Phylogroups, Pathotypes and Antibiotic Resistance Profiles of E. coli Isolates from Freshwater and Wastewater in the City of Panama
Source: Pathogens. 2025 Jun 20;14(7):617. doi: 10.3390/pathogens14070617 (PMC12299560; doi:10.3390/pathogens14070617)
Supplement: Supplementary file 1 [file pathogens-14-00617-s001.zip › pathogens-3629774-supplementary.pdf]

Supplementary Material

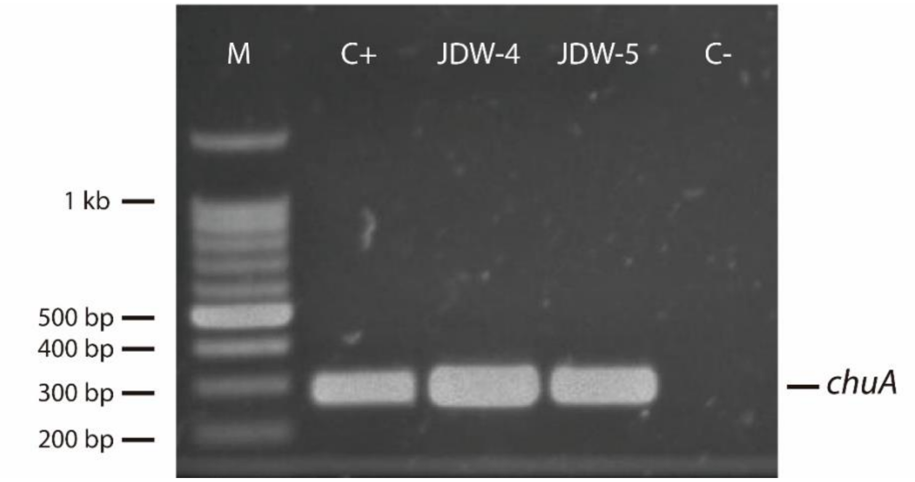

Supplementary Figure S1. Detection of *chuA* gene. Lane 1: molecular marker (M), lane 2: positive control (C+), lanes 3-4: positive samples, lane 5: negative control (C-).

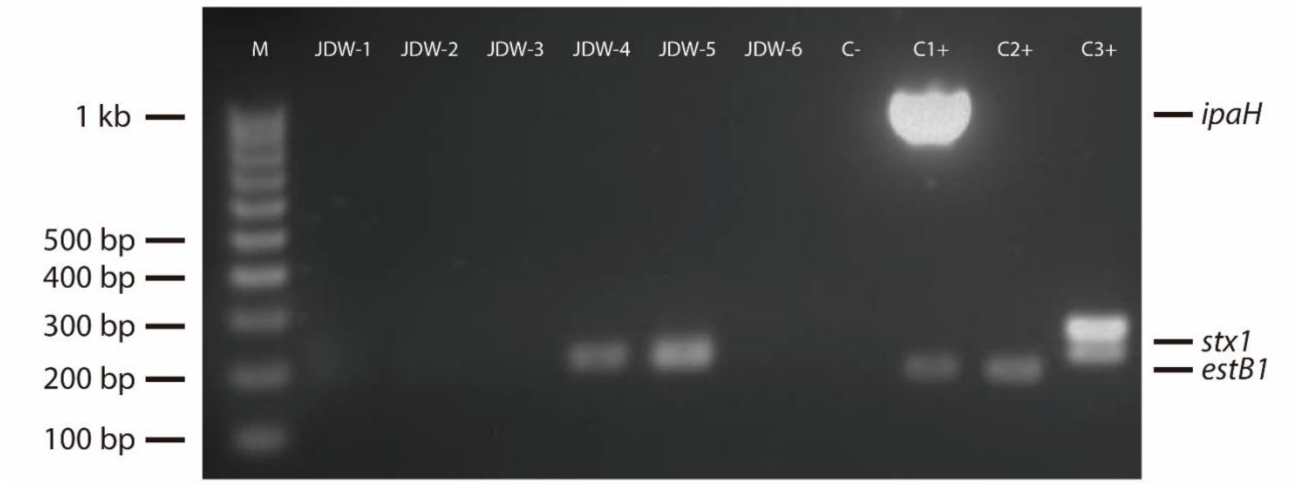

Supplementary Figure S2. Results of Multiplex PCR 2 for detection of EHEC, EIEC and ETEC pathotypes. Lane 1: molecular marker (M), lanes 2-7: samples, lane 8: negative control (C-) and lanes 9-11: positive controls (C+) for *ipaH*, *estB1*, and e*stB1*/*stx1* genes, respectively.

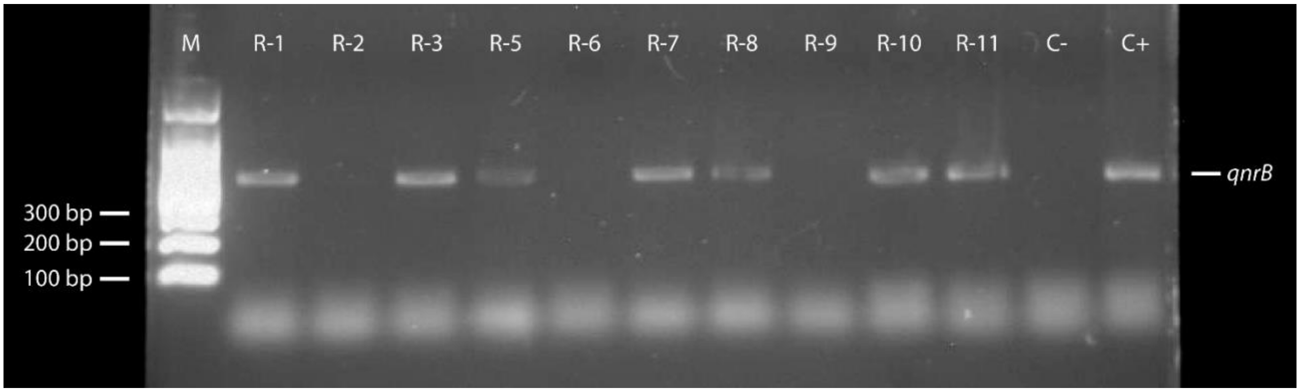

Supplementary Figure S3. PCR detection of *qnrB* group. Lane 1: molecular marker (M), lane 2-11: samples, lane 12: negative control (C-) and lane 13: positive control (C+).
